# Relating Urban Biodiversity to Human Health With the ‘Holobiont’ Concept

**DOI:** 10.3389/fmicb.2019.00550

**Published:** 2019-03-26

**Authors:** Jacob G. Mills, Justin D. Brookes, Nicholas J. C. Gellie, Craig Liddicoat, Andrew J. Lowe, Harrison R. Sydnor, Torsten Thomas, Philip Weinstein, Laura S. Weyrich, Martin F. Breed

**Affiliations:** ^1^School of Biological Sciences, The Environment Institute, The University of Adelaide, Adelaide, SA, Australia; ^2^Centre for Marine Bio-Innovation (CMB), School of Biological, Earth and Environmental Sciences, The University of New South Wales, Sydney, NSW, Australia; ^3^Australian Centre for Ancient DNA, School of Biological Sciences, The University of Adelaide, Adelaide, SA, Australia

**Keywords:** ecosystem services, immune, holobiont, microbiome, non-communicable disease, restoration ecology, biophilic cities, urban

## Abstract

A relatively unaccounted ecosystem service from biodiversity is the benefit to human health via symbiotic microbiota from our environment. This benefit occurs because humans evolved alongside microbes and have been constantly exposed to diverse microbiota. Plants and animals, including humans, are organised as a host with symbiotic microbiota, whose collective genome and life history form a single holobiont. As such, there are interdependencies between biodiversity, holobionts, and public health which lead us to argue that human health outcomes could be improved by increasing contact with biodiversity in an urban context. We propose that humans, like all holobionts, likely require a diverse microbial habitat to appropriate resources for living healthy, long lives. We discuss how industrial urbanisation likely disrupts the symbiosis between microbiota and their hosts, leading to negative health outcomes. The industrialised urban habitat is low in macro and microbial biodiversity and discourages contact with beneficial environmental microbiota. These habitat factors, alongside diet, antibiotics, and others, are associated with the epidemic of non-communicable diseases in these societies. We suggest that restoration of urban microbial biodiversity and micro-ecological processes through microbiome rewilding can benefit holobiont health and aid in treating the urban non-communicable disease epidemic. Further, we identify research gaps and some solutions to economic and strategic hurdles in applying microbiome rewilding into daily urban life.

## Introduction

The concept of holobionts (see Supplementary Material for glossary) encompasses the coevolution of complex life and microbiota in a microbial world. A holobiont is a host and its microbiota, which together form an individual with a metagenome under natural selection (Turnbaugh et al., 2007) (c.f. the hologenome theory of evolution, Zilber-Rosenberg and Rosenberg, 2008). Indeed, microbiota are essential to many biological systems and processes. These processes are diverse and include phytohormone production in plants (Friesen et al., 2011) and immunomodulation in animals, including humans (Rook et al., 2003).

Microbiota have key roles in forming holobionts while also providing and supporting multiple ecosystem services that benefit human health. Indeed, resilience of ecosystems and improvement in public health are increased by greater biodiversity, which is important to consider during rapid global change (WHO, 2015). It is therefore of no surprise that people who live in more biodiverse environments and with better access to parks and large green spaces become ill less often and live longer than those who live in less biodiverse areas, regardless of socioeconomic status (Evans, 2003; Brindley et al., 2018). These health benefits likely result from, in part, exposure to a rich source of microbiota to fulfil the needs of holobionts and are provided by biodiverse environments (Sarukhan et al., 2005).

**FIGURE 1:**
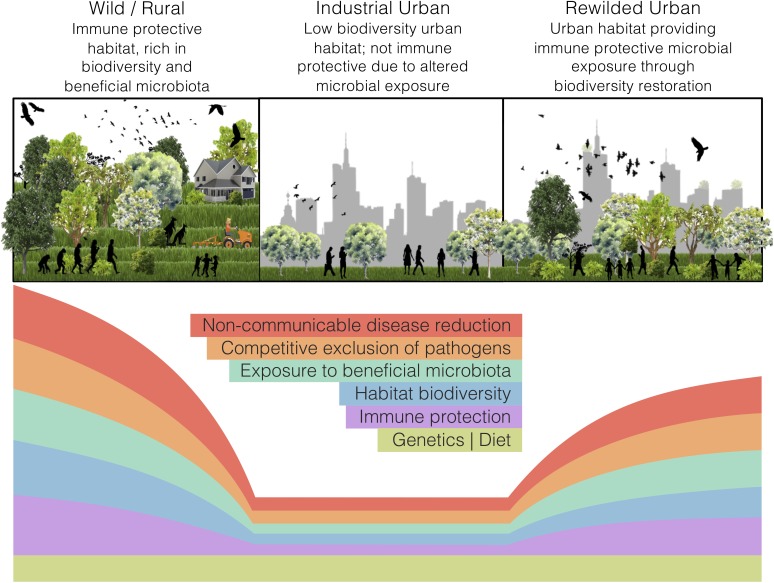
The microbiome rewilding hypothesis proposes the return of human habitat to one high in microbial diversity and with wilder symbiotic, competitive, and predatory micro-ecological processes. States of human habitat have varied levels of biodiversity and microbiota exposure, immune protection, microbial processes, and non-communicable disease rates. Genetics and diet can remain the same across habitat states.

Land-use change, such as industrial urbanisation (proceeding mentions of ‘urban’ refer to ‘industrialised urban’), alters the exposure of inhabitants to natural habitats. In a microbial world, such alterations may interfere with the microbial colonisation of a host and disrupt holobiont development. Our perspective article focuses on the impacts of land-use change, specifically urbanisation, on the interdependencies of ecosystems, holobionts, and non-communicable disease. We argue that urban health issues can be somewhat alleviated through microbiome rewilding – the restoration of microbial biodiversity in urban areas (Figure 1) – and discuss an initiative that aligns with this way of thinking. Additionally, we highlight possible solutions for economic and strategic hurdles that need to be addressed to implement microbiome rewilding as a preventative urban health intervention (Mills et al., 2017).

## Holobionts Are Formed Through Environmental Interactions Providing Health Benefits

**FIGURE 2:**
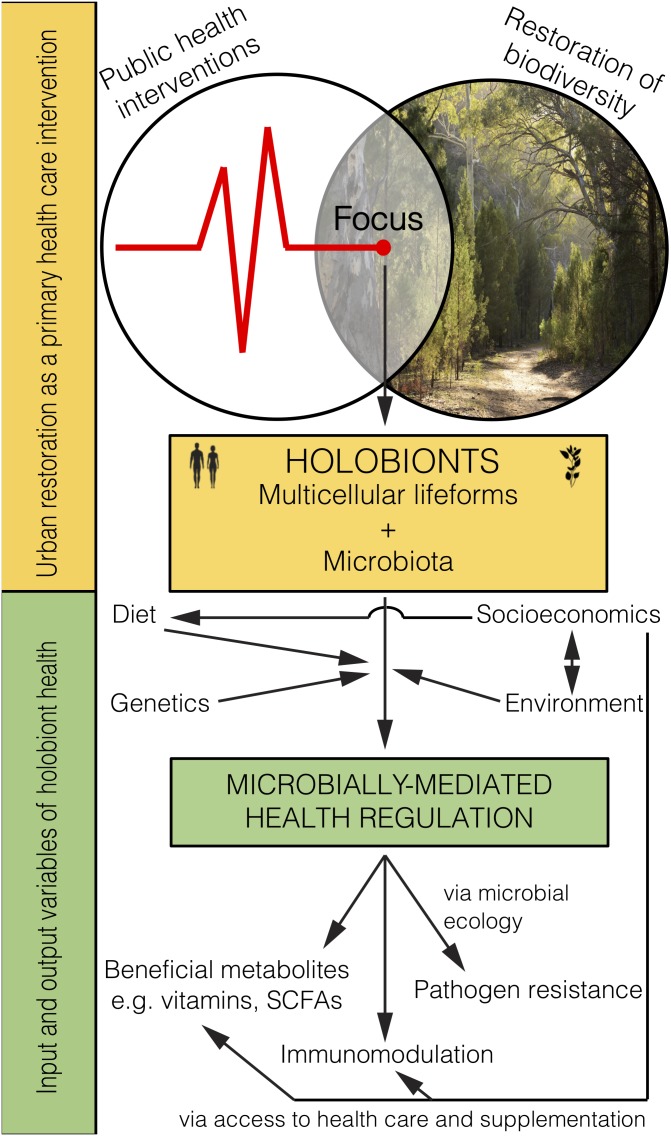
Microbiome rewilding via the restoration of biodiversity to urban habitats holds great potential as an environmental input to microbially-mediated health regulation of holobionts, including humans, as a primary health intervention that transcends socioeconomic status.

Holobiont microbiota are determined by many factors that influence their colonisation of a host, including genetics, lifestyle, and environmental interactions (Rook et al., 2014). Initial microbial colonisation has been described under the ecological theory of succession (Costello et al., 2012). For example, the primary succession of a child is tumultuous in early-life. As children gather immunomodulatory microbiota their immune systems learn to properly respond to the diversity of environmental inputs (Figure 2). The gut microbiota eventually stabilise around the age of three (Grice et al., 2009) and this early-life colonisation appears most critical for immune training and health outcomes (Gilbert et al., 2018). However, microbiota are susceptible to disturbance, such as medical treatment (e.g., antibiotics) or large changes to lifestyle (e.g., severe dietary change; David et al., 2014). Additionally, pathogens are subject to ecological processes such as competitive exclusion and predation. Several known pathogens, including *Bordetella* spp., use immune-mediated competition (Weyrich et al., 2012), while others, such as *Vibrio* spp., use direct competition with the host microbiota (Unterweger et al., 2014). Succession principles play key roles in establishing and maintaining healthy microbiota. Altering these principles, especially in early-life, can have detrimental health outcomes.

Health outcomes are thought to be improved by the immune protective effects of biodiverse environments via coevolved microbiota. For example, microbiome differences were observed between the Amish (manual agriculturalists) and the Hutterites (mechanised agriculturalists), which associated with greater immune protection in the Amish (Stein et al., 2016). Further, urban children, whose microbiomes differ to those of rural children, are more susceptible to asthma compared to farm children, who are apparently also protected from allergies by the microbiome of their house dust (Hanski et al., 2012; Schuijs et al., 2015; Birzele et al., 2017). Also, an Australia-wide study suggests biodiversity has a protective role for respiratory health (Liddicoat et al., 2018b).

Greater contact with environmental microbiota may also be protective against infectious disease. Environmental microbiota supplement our own protective microbiota, participate in immune signalling (promoting both inflammatory and tolerance-inducing responses), and help build adaptive immunity. Together, these factors contribute to immune fitness against infectious disease (von Hertzen et al., 2011; Molloy et al., 2012; Rook et al., 2014). For example, bacteria can play a key role in controlling adaptive immunity against a viral respiratory infection (Ichinohe et al., 2011). Also, reduced rates of infectious and parasitic disease were found in populations surrounded by high cation exchange capacity soils, which generally support high microbial diversity (Liddicoat et al., 2018a).

## Ecosystem Impacts, Urban Habitats, and Disease: the Case for Microbiome Rewilding

### Impacted Ecosystems Cause Public Health Issues

Healthy ecosystems and their resident macro-organisms have a microbiota that is moderated by ecological processes, such as resource availability and competition. Further, environmental microbiota are key components of many ecosystem processes, such as nutrient cycling and water filtration. However, urbanisation can impact on these processes and allow certain taxa to thrive or be diminished, sometimes resulting in important consequences for human health. For example, eutrophic urban waterways combined with altered water flows can allow cyanobacteria to bloom. Eutrophication occurs when excess nutrients (e.g., fertiliser) run-off from lawns, gardens, and decaying leaf litter (Wallace et al., 2008) into waterways, priming for bloom conditions. Cyanobacteria can produce a suite of hepatotoxins and neurotoxins, which have been associated with health issues, including tumour promotion and liver failure (Falconer, 1991; Falconer et al., 1994; Cheung et al., 2013). Moreover, the cyanotoxins implicated in disease find their way into the diets of many holobionts, including humans, through bioaccumulation in fishery species (Ibelings and Chorus, 2007; Scott et al., 2018). Many non-communicable diseases are associated with impacted habitats which likely then degrades the ecosystem that is a holobiont.

### Habitat Change From Urbanisation Strongly Associates With Disease

Mammalian-holobionts are walking ecosystems with coevolved, yet distinct, microbiota (Nelson, 2015; Robinson et al., 2018; Ross et al., 2018), and differences in their microbiota relate to differentiation in genetics, diet, lifestyle, and habitat. Microbiota that colonise, or simply pass through, from the environment are important to mammalian health because they influence immunomodulatory development in early-life. Therefore, when mammalian habitats change, such as when raised in captivity, microbiota and immune function are also altered. For instance, Mexican black howler monkeys, *Alouatta pigra*, living in degraded or captive habitat had less diverse gut microbiomes compared to those in natural habitats (Amato et al., 2013). Additionally, *A. pigra* living in the non-natural habitats had less diverse diets and reduced abundances of microbial genes related to metabolism and immune function. Another study looked at the microbiome and immune development in piglets born indoors vs. outdoors, which showed those raised indoors had relatively low microbial diversity (Mulder et al., 2011). Piglets born outdoors also had more normal microbially-mediated immune function in early-life, but this difference reduced over time as all piglets were subsequently raised in a high-hygiene facility.

Domestication has also influenced the microbiota and health of mammals. The gut microbiome of a domesticated horse species*, Equus ferus* ssp. *caballus*, was less diverse than the world’s only undomesticated horse species, *E. ferus* ssp. *przewalskii*, living in nearby native grasslands (Metcalf et al., 2017). In a study of domestic dogs, those from urban areas had more allergies and a more human-like skin microbiome than rural dogs, who carried more environmental microbiota (Lehtimäki et al., 2018). Furthermore, domesticated animals also suffer non-communicable diseases common to urban people (Jensen-Jarolim, 2017). In addition, most animal species placed in zoos with a ‘domesticated’ lifestyle also maintain lower diversity and distinct gut microbiomes compared to wild counterparts (Muegge et al., 2011). The impacts of lifestyle and habitat changes on mammalian-holobiont disease states are also found in humans.

Industrialised cities continue to see global rises in many human non-communicable diseases despite advances in modern medicine (Ehlers et al., 2010). This rise has been linked to modern lifestyles and the urban environments and microbial exposures in industrialised countries (Rook et al., 2003; von Hertzen et al., 2011). Similarly, many non-communicable immune diseases are linked to altered microbial community structure in the gut, and include childhood asthma (Depner et al., 2017), multiple sclerosis (Berer et al., 2017), some cancers (Ahn et al., 2013; Schwabe and Jobin, 2013), diabetes (Karlsson et al., 2013), and many others (reviewed in Cho and Blaser, 2012). Indeed, studies in mouse models have begun to mechanistically link altered microbiomes to non-communicable, inflammatory diseases (Turnbaugh et al., 2006; Schrumpf et al., 2017). These microbiome alterations change the immune profile of unhealthy individuals, increasing their inflammation and resulting in disease.

Further, the risk of developing inflammatory bowel disease (IBD) is increased for industrial urban residents (Loftus et al., 2009; Cholapranee and Ananthakrishnan, 2016; Paramsothy et al., 2017). This trend indicates that the IBD burden is, to some degree, associated with inadequate exposure to biodiversity. For example, ownership of cats and dogs which carry outdoor microbiota, and exposure to farms which contain large microbial diversities, are both negatively associated with altered microbiota and IBD (Radon et al., 2007), suggesting that both domestic and environmental biodiversity are immune protective. However, while IBD is tightly linked with altered gut microbiota, it is still unclear whether this alteration is pathological or symptomatic. Although routine early-life exposure to biodiverse environments is likely to have a positive health influence on humans via their immunomodulatory microbiota.

Restoring biodiverse habitat for animals and people in urban areas should lead to the improved health of those exposed (Figure 2). Importantly, it remains to be tested whether and how the human microbiome changes from interactions with high biodiversity urban areas, and whether such interactions lead to positive health outcomes. Indeed, early-life interactions with rewilded or agricultural urban spaces and domesticated animals may be the most critical time for positive health outcomes. Even though the microbiota obtained from animals and the environment are not likely to be adapted for living within the human body (e.g., non-human parasites, soil bacteria) they would provide a fleeting immune challenge and subsequent immune training. Furthermore, as plant communities are restored, food webs will become diversified with important implications for the environmental microbiota that help promote and support the increasing diversity of plants and animals.

### Importance of Considering Risks of Microbiome Rewilding in Context

Microbiome rewilding of urban areas potentially comes with increased risks, such as interactions with dangerous animals and zoonotic diseases. Moreover, increased vegetation structure may increase the risk of falling branches, while poorly designed green spaces may be more flammable (e.g., if dominated by eucalypts or pines). Factors like these highlight the need for well-designed green spaces with flora and fauna management, especially when these high biodiversity green spaces are set among high-density urban residences. However, there is a trade-off between these risks and the risks of maintaining traditional urban habitats.

Public health risks come with maintaining traditional urban habitats. For example, the previously mentioned cyanobacteria growing unchecked in eutrophic water exude dissolved organic carbon as they grow and die. Organic carbon reacts with chlorine – added as a disinfectant during water treatment to eliminate microbial pathogens – forming halogenated organic compounds (Tomlinson et al., 2016). These compounds, collectively referred to as disinfection by-products, can reach problem levels when algal abundance is high in the source water and when there is a high dissolved organic carbon load exported from the catchment. Health risks linked with disinfection by-product exposure include the potential association with bladder cancer, as well as links to miscarriages and birth defects (Hrudey, 2009; Thomson et al., 2014). Promoting native biodiversity, that leaches less nutrients from leaf litter (Wallace et al., 2008) with less fertiliser input and run-off, could reduce waterway eutrophication and toxic blooms. Therefore, restoring urban green spaces with native and biodiverse plant communities could help prevent disease by absorbing and sequestering nutrients. Overall, functionally intact ecosystems should support public health by regulating both free-living and symbiotic microbiota, which feedback to the overall health of ecosystems. However, research on the risks and benefits of rewilding functional and microbially-rich ecosystems in urban contexts remains limited.

## Plant-Holobionts: the Primary Microbiome Rewilders

Diverse plant communities attract and support microbial and animal diversity, such as insects, birds, and mammals, which are themselves holobionts and promoters of microbial diversity. These plant communities have long played a major role in the basis of healthy mammalian habitats, however, they are heavily impacted by urbanisation. Therefore, we will explore the plant-holobiont to demonstrate their key role in rewilding micro-ecological processes to urban habitats.

Microbiota and plants have well-studied coevolutionary relationships (Bonkowski et al., 2009; Van Nuland et al., 2016). For example, many plants use a two-step selection process to filter their rhizosphere microbiota from high diversity bulk soil (Bulgarelli et al., 2013; Philippot et al., 2013). First, plants exude photosynthates – an attractive source of carbon – that influence microbial exchange from bulk soil to rhizosphere (Bonkowski et al., 2009; Berendsen et al., 2012). These photosynthates can support a core rhizosphere microbiome, often with lower diversity than surrounding bulk soil (Bulgarelli et al., 2012; Urbina et al., 2018). Secondly, microbes filter internally from the root surface, again with decreased diversity. Microbes that are adapted to this internal environment are selected by host-specific mechanisms. In addition, generalist saprotrophic taxa are often enriched inside roots, which contain dead woody material (Bulgarelli et al., 2012; Urbina et al., 2018). Complex interactions such as this likely lead to covariation between plant and microbial communities.

This covariation between plant and microbial communities is likely explained by feedback loops between plant traits and their environment. In these loops, symbiotic microbiota are both a driver and a responder, which influence and are influenced by plant traits (Rosado et al., 2018). For example, in a long-term vegetation community trial, plant species richness, plant functional identity, and plant community functional diversity explained 41% of leaf surface microbiome structure, while leaf microbial diversity explained a large amount of variation in plant community productivity (Laforest-Lapointe et al., 2017). Additionally, microbiota can be powerful mediators of plant functional traits (Vandenkoornhuyse et al., 2015). They can synthesise bioactive compounds that plants cannot, while also producing many phytohormones (Friesen et al., 2011). Studies also indicate that endo- and epiphytic microbiota have functional roles in plants, such as influencing water retention and nitrogen fixation on leaf surfaces (Beattie, 2011; Moyes et al., 2016). Additionally, an alteration of symbiotic microbiota can impede plant growth (Wubs et al., 2016). Further, leaf microbial diversity can improve plant health by providing resilience to pathogens through competitive exclusion (Ritpitakphong et al., 2016).

As such, the plant-holobiont has a strong influence on its environmental microbiota, as well as being a primary producer in food webs. Correlated succession of environmental microbiota and plant communities follow natural disturbances and ecological restoration (Rime et al., 2015; Gellie et al., 2017; Yan et al., 2018). Therefore, restoration of diverse plant communities in urban areas may provide human health benefits by diversifying the environmental microbiota. To provide these health benefits, future work needs to focus on how to design, restore, and manage urban green spaces to optimise microbial exposure (Mills et al., 2017; Robinson et al., 2018).

## Microbiome Rewilding – From Hypothesis Into Practise

An important issue in the restoration economy is that projects are predominantly accounted for in market terms, such as carbon, water, and timber products (De Groot et al., 2013). These products do not accurately reflect non-market financial benefits. For example, urban restoration projects may provide several direct and indirect health benefits (e.g., green space for exercise; microbially-mediated health benefits), yet these are not well considered or quantified. Such restoration projects may not fall within traditional restoration ecology, but incorporate values of ‘microbiome-inspired green infrastructure’ (MIGI) that influence the microbial exposure of urban residents while incorporating co-benefits (Robinson et al., 2018). Co-benefits of incorporating MIGI to urban areas may include spaces for food foraging or urban community gardens, which come with dietary benefits, and green walls with diverse flora, fauna, and microbiota, which can also intercept air and noise pollution.

However, restoration projects face financial challenges. For example, the net return of achieving the Bonn Challenge of restoring 350 million hectares of degraded land by 2030 has recently been estimated at $USD 2-to-9 trillion in ecosystem services over 50 years (Verdone and Seidl, 2017). This return would seem to be a legitimate incentive for investment, particularly in and around urban areas. Yet, the Bonn Challenge remains underfunded (Ding et al., 2017), illustrating the need for reform on how restoration projects are accounted for and financed. An opportunity exists in overcoming some of these funding shortfalls by consciously aligning the restoration and health economies; rewilding urban microbiota through restoration should provide direct human health benefits and public health cost savings (Mills et al., 2017). As such, restoration projects that aim to align the co-benefits of microbiome rewilding (e.g., air filtration, heat-island mitigation, human health) should actively look for synergies by working in partnership with health sectors and local governments. For example, the Healthy Urban Microbiome Initiative (HUMI) aims to develop urban restoration projects around the world that incorporate the interests of communities who have needs, local governments who want to maximise co-benefits, and health sectors who oversee disease management (for more information see https://www.humi.site/ and Flies et al., 2018). Combining these three interests under HUMI should maximise the potential economic benefits of microbiome rewilding by leveraging funds for each interest group. Indeed, the absence of aligning these interests and not funding interventions may be costly.

It is estimated that where health interventions are not implemented, non-communicable diseases (such as asthma, atopic allergies, inflammatory bowel disease) will cost low- and middle-income countries $USD 7 trillion for the period 2011–2025 (WHO, 2014). Hypothetically, that cost could be reduced by $USD 350 billion if the relatively low-cost intervention of microbiome rewilding reduces non-communicable diseases by just 5%. Using inflammatory bowel disease (IBD) as an example of health costs associated with a potential microbial exposure deficit, we can estimate a fraction of the costs that restoration could recover. As of 2013, the European Union was spending €4.6 to 5.6 billion annually on IBD treatment (Ganz et al., 2016). Therefore, if urban restoration can reduce health care costs by 5% then the European Union could save €230-to-280 million per annum on IBD alone.

The health of holobionts, including humans, is fundamentally linked to the health of ecosystems and potentially driven by the state of environmental microbiota. While further work is needed on the specific causative mechanisms underpinning these positive health associations, restoring urban biodiversity provides a low risk and low-cost investment that is likely to rewild urban microbial processes and have potential to pay generational health dividends. We believe that these restoration investments are imperative for tackling the ongoing decline in urban green spaces, rapid growth in urban populations, and increases in microbially-mediated non-communicable diseases.

## Author Contributions

JM wrote the first draft and all authors contributed substantially to the text and editing.

## Conflict of Interest Statement

The authors declare that the research was conducted in the absence of any commercial or financial relationships that could be construed as a potential conflict of interest.
