# Supplementary material for: Relating Urban Biodiversity to Human Health With the ‘Holobiont’ Concept
Source: Front Microbiol. 2019 Mar 26;10:550. doi: 10.3389/fmicb.2019.00550 (PMC6444116; doi:10.3389/fmicb.2019.00550)
Supplement: Supplementary file 1 [file Table_1.pdf]

Supplementary Material

Relating urban biodiversity to human health with the 'holobiont' concept

Jacob G. Mills*, Justin D. Brookes, Nicholas J.C. Gellie, Craig Liddicoat, Andrew J. Lowe, Harrison R. Sydnor, Torsten Thomas, Philip Weinstein, Laura S. Weyrich, Martin F. Breed

***Correspondence:** Jacob G. Mills, millsj515@gmail.com

1 Glossary of terms

Adaptive immunity Immunity generated in response to exposure to a new antigen, and which will respond specifically to that antigen on subsequent exposures.

Bioaccumulation Accumulation of environmental substances in organisms, which are often due to bottom-up effects in trophic systems.

Bioactive compounds Compound that influences the activity of an organism, e.g. flavonoids, vitamins.

Bulk soil Soil beyond the rhizosphere.

Cation exchange capacity Overall capacity of soil particles to hold exchangeable cations.

Competitive exclusion (a.k.a. Gause's Law) Two species that compete for identical resources cannot stably coexist, and therefore in a diverse environment an invader will find collective competition by the community for all of its required resources.

Cyanotoxins Toxins produced by cyanobacteria, including hepatotoxins and neurotoxins.

Direct competition Direct competition for resources between organisms.

Disinfection by-products Products that result from the reaction of organic and inorganic matter with water-treatment chemicals.

Domestication The process of taming or selective-breeding an organism or species to suit human use.

Ecosystem services Social, economic, and environmental benefits of ecosystems which fall into four categories; *provisioning*, e.g. water, food, fuel; *regulating*, e.g. climate, health; *supporting*, e.g. nutrient cycling, oxygen production; and *culture*, e.g. sense of place, recreation.

Endophytic Living within plant tissue.

Epiphytic Living on plant tissue.

Eutrophic A water body rich enough in nutrients that it supports large plant or microbial populations, which can then create anoxic conditions that can potentially kill animal life.

Halogenated organic compounds Class of natural and synthetic chemicals containing at least one halogen (chlorine, fluorine, bromine, iodine).

Hepatotoxins Toxins that damage the liver.

Holobiont A multicellular host and its symbiotic microbiota, which together form an evolutionary individual with a collective genome; a.k.a metaorganism, supra-organism.

Immune protection Where environmental conditions (e.g. due to high microbial diversity) tend to promote immune fitness or adequate immune function within resident populations.

Immune signalling Activation of specific immune activities.

Immune-mediated competition Where the effects of the immune system give a microbe an indirect competitive advantage by suppressing its competitors.

Immunomodulation Education and regulatory adjustment of the immune system.

Infectious disease Disease caused by a transmittable organism or genetic material (e.g. viruses).

Inflammation Immune reaction in an attempt to control harmful stimuli, such as a foreign body, which can result in swelling, redness, heat, and pain.

Inflammatory bowel disease Group of diseases causing inflammation of the colon and small intestine; Crohn's disease and ulcerative colitis are the most notable types.

Microbiome Collective genomic material of microorganisms in a defined environment.

Microbiome Rewilding Restoration of diverse microbiota to human habitats, provisioned by biodiversity, with potential health benefits through enhancement of immunomodulatory, metabolic, endocrine, and other microbiota-mediated processes.

Microbiota An ecological community of microorganisms within a defined environment (e.g. gut microbiota).

Neurotoxins Toxins that affect the nervous system.

Nitrogen fixation Process of converting atmospheric nitrogen into bioavailable ammonium.

Non-communicable disease Diseases not caused by infectious agents, such as chronic inflammatory diseases.

Photosynthates Products of photosynthesis.

Phytohormone Plant hormones (e.g. auxins, gibberellins) produced by plants and their microbiota involved in metabolism, growth, or other physiological activities of the plant.

Rhizosphere The region surrounding the root, which is chemically and microbially influenced by the plant's activity.

Saprotrophic Process of extracellular digestion of dead or waste organic matter.

Succession Accumulation and turnover of species in an environment over time, where primary succession occurs in a novel environment (e.g. glacial fore-field, infant) and secondary succession occurs in a disturbed environment (e.g. cleared forest, antibiotic treatment).

Symbiotic microbiota Mutualistic, commensal, parasitic, and pathogenic microbiota of a supra-organism.

Urbanisation Where populations transfer from rural to urban areas with a proportional increase in urban residents, and the differentiation of rural and urban societies.

Costello, E.K., Stagaman, K., Dethlefsen, L., Bohannan, B.J., and Relman, D.A. (2012). The application of ecological theory toward an understanding of the human microbiome. *Science* 336**,** 1255-1262.

Ross, A.A., Müller, K.M., Weese, J.S., and Neufeld, J.D. (2018). Comprehensive skin microbiome analysis reveals the uniqueness of human skin and evidence for phylosymbiosis within the class Mammalia. *Proceedings of the National Academy of Sciences* 115**,** E5786-E5795.
